# Air Bubbles Activate Complement and Trigger Hemostasis and C3-Dependent Cytokine Release Ex Vivo in Human Whole Blood

**DOI:** 10.4049/jimmunol.2100308

**Published:** 2021-12-01

**Authors:** Benjamin S. Storm, Dorte Christiansen, Hilde Fure, Judith K. Ludviksen, Corinna Lau, John D. Lambris, Trent M. Woodruff, Ole-Lars Brekke, Tonje Braaten, Erik W. Nielsen, Tom Eirik Mollnes

**Affiliations:** * Department of Anesthesia and Intensive Care Medicine, Surgical Clinic, Nordland Hospital, Bodø, Norway;; † Institute of Clinical Medicine, UiT The Arctic University of Norway, Tromsø, Norway;; ‡ Faculty of Nursing and Health Sciences, Nord University, Bodø, Norway;; § Research Laboratory, Nordland Hospital Trust, Bodø, Norway;; ¶ Perelman School of Medicine, University of Pennsylvania, Philadelphia, PA;; ‖ School of Biomedical Sciences, Faculty of Medicine, The University of Queensland, St. Lucia, Queensland, Australia;; # Department of Community Medicine, UiT The Arctic University of Norway, Tromsø, Norway;; ** Faculty of Medicine, Institute of Clinical Medicine, University of Oslo, Oslo, Norway;; †† Faculty of Health Sciences, K.G. Jebsen Thrombosis Research and Expertise Center, UiT The Arctic University of Norway, Tromsø, Norway;; ‡‡ Department of Immunology, Oslo University Hospital and the University of Oslo, Oslo, Norway; and; §§ Centre of Molecular Inflammation Research, Norwegian University of Science and Technology, Trondheim, Norway

## Abstract

Venous air embolism, which may complicate medical and surgical procedures, activates complement and triggers thromboinflammation. In lepirudin-anticoagulated human whole blood, we examined the effect of air bubbles on complement and its role in thromboinflammation. Whole blood from 16 donors was incubated with air bubbles without or with inhibitors of C3, C5, C5aR1, or CD14. Complement activation, hemostasis, and cytokine release were measured using ELISA and quantitative PCR. Compared with no air, incubating blood with air bubbles increased, on average, C3a 6.5-fold, C3bc 6-fold, C3bBbP 3.7-fold, C5a 4.6-fold, terminal complement complex sC5b9 3.6-fold, prothrombin fragments 1+2 (PTF1+2) 25-fold, tissue factor mRNA (TF-mRNA) 26-fold, microparticle tissue factor 6.1-fold, β-thromboglobulin 26-fold (all *p* < 0.05), and 25 cytokines 11-fold (range, 1.5–78-fold; all *p* < 0.0001). C3 inhibition attenuated complement and reduced PTF1+2 2-fold, TF-mRNA 5.4-fold, microparticle tissue factor 2-fold, and the 25 cytokines 2.7-fold (range, 1.4–4.9-fold; all *p* < 0.05). C5 inhibition reduced PTF1+2 2-fold and TF-mRNA 12-fold (all *p* < 0.05). C5 or CD14 inhibition alone reduced three cytokines, including IL-1β (*p* = 0.02 and *p* = 0.03). Combined C3 and CD14 inhibition reduced all cytokines 3.9-fold (range, 1.3–9.5-fold; *p* < 0.003) and was most pronounced for IL-1β (3.2- versus 6.4-fold), IL-6 (2.5- versus 9.3-fold), IL-8 (4.9- versus 8.6-fold), and IFN-γ (5- versus 9.5-fold). Antifoam activated complement and was avoided. PTF1+2 was generated in whole blood but not in plasma. In summary, air bubbles activated complement and triggered a C3-driven thromboinflammation. C3 inhibition reduced all mediators, whereas C5 inhibition reduced only TF-mRNA. Combined C5 and CD14 inhibition reduced IL-1β release. These data have implications for future mechanistic studies and possible pharmacological interventions in patients with air embolism.

## Key Points

- Air bubbles trigger a C3-driven thromboinflammation in human whole blood.
- Blocking C3, but not C5, attenuates the air-induced inflammation.
- Avoiding ambient air in test tubes attenuates thromboinflammation.

## Introduction

Venous air embolism may complicate many surgical and medical procedures, including vascular access, open or laparoscopic procedures, biopsies, interventional radiological procedures, and extracorporeal blood circulations such as dialysis, plasmapheresis, or heart and lung machine circulation (1, 2). Air emboli can cause acute hemodynamic collapse and trigger life-threatening inflammatory lung edema (3, 4), with no established specific anti-inflammatory treatment.

The central C3 molecule in the complement system contains an internal thioester bond making C3b able to bind covalently to solid surfaces after cleavage of C3 (5, 6). Low-grade spontaneous hydrolysis of this thioester bond occurs in the fluid phase, changing C3 to C3(H_2_O), also termed “iC3” or “C3b-like,” where C3a is not cleaved off (7–9). This molecule binds factor B, which is cleaved by factor D, and the initial alternative pathway C3 convertase, C3(H_2_O)Bb, is generated and cleaves C3 into C3a and C3b. In vitro, in antifoam-treated human serum and heparin-anticoagulated whole blood, it has been shown that bubbles of ambient air, nitrogen, and oxygen equally triggered the change in C3 configuration to iC3 (10). Furthermore, bubbles of nitrogen, helium, argon, and ambient air equally activated platelets (11). Together, these studies indicate that the gas–plasma interface per se rather than a specific gas composition initiates the complement activation. Whether the C3 activation could trigger further downstream activation, including activation of C5, was not investigated in these studies.

In vivo animal studies have shown that air emboli can trigger inflammation (12, 13), activate platelets, and cause thrombocytopenia and coagulopathy (14). An intimate interaction exists between the complement, hemostatic, and inflammation systems, and activation of one system may trigger the others (15–18). The role of the alternative complement pathway in these interactions has been extensively reviewed (19). C3 and C5 may be pivotal for this interaction, because C3a and C5a can bind to their receptors, C3aR, C5aR1, and C5aR2, and activate many cells, including platelets, leukocytes, and endothelial cells (17, 20, 21). In the present ex vivo study, we used lepirudin-anticoagulated human whole blood without ambient air in the test tubes (22, 23) to study the effect of air embolism, mimicked by air bubbles, on the complement, hemostatic, and cytokine systems. In this paper, we use the term “coagulation” when discussing the coagulation system and the term “hemostasis” when discussing the joint effect of platelet activation, tissue factor, and coagulation.

Lepirudin (recombinant hirudin) is a highly specific thrombin inhibitor that blocks coagulation at the last step before clotting, leaving other cascade systems and inflammatory networks uninhibited and open for mutual interaction (23). In contrast, most other anticoagulants, such as EDTA, citrate, and heparin, interfere with several plasma cascades and cell functions, and they are best avoided when studying the inflammatory responses. Thus, lepirudin anticoagulated whole blood represents the most physiologically relevant ex vivo human model available, to our knowledge.

Antifoam is often used to reduce foam formation in experiments with air bubbles. However, little attention has been paid, first, to whether antifoam may have adverse effects on the inflammatory readouts and, second, whether antifoam is needed for performing such experiments. Thus, we designed experiments to answer these questions.

The primary aim of this study was to investigate the extent to which complement activation contributes to the thromboinflammatory reaction induced by air bubbles. Our three secondary aims were to examine the effect of air bubbles on physiologic parameters, including partial pressure of oxygen (pO_2_), partial pressure of carbon dioxide (pCO_2_), and pH; the effect of antifoam reagents on the complement and coagulation systems; and the effects of air bubbles on complement and coagulation in whole blood compared with plasma.

## Materials and Methods

**FIGURE 1.**
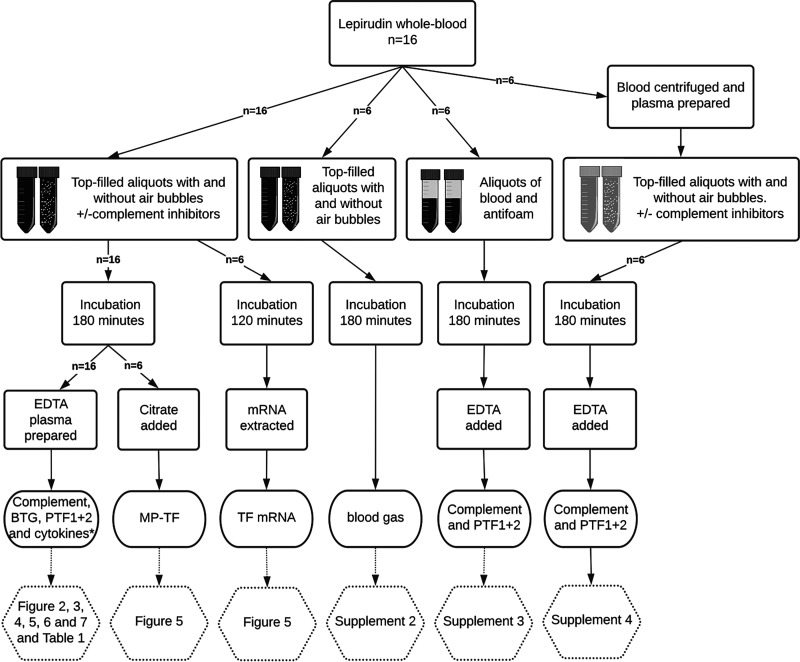
Study design. Lepirudin blood and plasma from 16 human donors were incubated in top-filled tubes with and without air bubbles and selected complement and inflammation inhibitors or with antifoam in half-filled tubes. Details of incubations, readouts, and references to figures are stated in the flowchart. *Number of samples included in analyses varies as described in *Materials Methods*.

A series of experiments were conducted (summarized in (Figure 1) using the whole blood model (23) with modifications as previously described (22). The study was approved by the Regional Committee for Medicine and Health Research Ethics.

### Whole-blood experiments

All disposables and solutions were endotoxin-free. Blood from 16 healthy human donors (7 males and 9 females, aged 30–60 y) was collected using the BD Vacutainer Eclipse blood collection system (Becton Dickinson) into Nunc polypropylene tubes (Nunc, Roskilde, Denmark) prefilled with lepirudin (Refludan; Hoechst, Frankfurt am Main, Germany) to a final concentration of 50 µg/ml blood.

After drawing, each donor’s blood was pooled in 50 ml polypropylene conical Falcon tubes (Corning, Tamaulipas, Mexico) preheated to 37°C. On a block heater set to 37°C, we added 858 µl blood from each donor to sets of 1.8-ml Nunc polypropylene tubes prefilled with specific inhibitors of the complement and the TLR systems diluted in sterile PBS with CaCl_2_ and MgCl_2_ (PBS) (Sigma-Aldrich, St. Louis, MO) to a final volume of 142 µl as follows: the C3 inhibitor peptide Cp40 (produced at the University of Pennsylvania as previously described [24]) to a final concentration of 20 µM, the recombinant humanized anti-C5 mAb eculizumab (Soliris; Alexion Pharmaceuticals GmbH, Zürich, Switzerland) to a final concentration of 100 µg/ml, the C5aR1 blocking cyclic peptide PMX53 (produced at the University of Queensland, St. Lucia, Australia, as previously described [25]) to a final concentration of 10 µM, the anti-human CD14 recombinant IgG2/4 Ab r18D11 (produced at Nordland Hospital, Bodø, Norway, as previously described [26]) to a final concentration of 15 µg/ml, a combination of Cp40 and r18D11 to a final concentration of 20 µM and 15 µg/ml, respectively, or tubes containing only PBS. Additionally, 3003 µl of blood from each donor was mixed with 497 μl PBS in 5 ml Nunc tubes and from there transferred to 1.8-ml tubes, filling the tubes and the lids completely (as shown in (Figure 1A of [22]). To ensure that adequate concentrations of eculizumab and PMX53 were used in our experiments, we titrated these inhibitors; because the C5 concentration in serum and plasma is relatively constant during ex vivo experiments, we tested the eculizumab effect in serum using the Wieslab Complement System Alternative Pathway Wielisa assay (Svar Life Science, Malmö, Sweden) (Supplemental Fig. 1A). In contrast, C5aR expression on the cell surfaces can vary considerably during an experiment. Thus, we tested the effect of PMX53 in whole blood incubated with air using β-thromboglobulin (BTG), microparticle tissue factor (MP-TF), and prothrombin fragments 1+2 (PTF1+2) as readouts (Supplemental Fig. 1B–D).

Except for the “baseline” and “no air” samples, we bubbled ∼2.5 ml of ambient air through the blood using a 21-gauge sterile needle and 20-ml syringe, resulting in a mixture of air bubbles and blood filling the tubes (as shown in (Figure 1C of [22]). All tubes except baseline were incubated on a Rock’n’Roller tube roller mixer (Labinco, Breda, the Netherlands) at 37°C for 180 min. To the baseline tubes, we added 14.6 μl EDTA to a final concentration of 10 mM and kept the tube on ice. After incubation, we transferred 1 ml blood from the no-air tubes to new 1.8-ml Nunc tubes and added EDTA to all incubated samples to a final concentration of 10 mM. We centrifuged the samples at 3000 *g* for 20 min at 4°C, transferred the plasma to 1.0-ml polypropylene Matrix 2D storage tubes (Thermo Fisher Scientific, Waltham, MA), and stored the samples at −80°C for later analysis. Immediately before incubation and after incubation, we transferred 225 μl blood from six of the donors to new tubes, added 25 μl citrate (Vacuette; Greiner Bio-One GmbH, Frickenhausen, Germany), centrifuged the samples at 1,500 *g* for 15 min at room temperature (RT), isolated and centrifuged the supernatant at 13,000 *g* for 2 min, and stored plasma at −80°C until analysis of MP-TF. Additionally, we prepared and incubated blood from six donors (three males and three females) as described above. After 120 min of incubation, 650 μl of blood was transferred to 5-ml Matrix polypropylene tubes (Thermo Fisher Scientific) containing 1.8 ml of PAXgene RNA stabilization solution (BD Biosciences, San Jose, CA). The tubes were carefully tilted 10 times, kept at RT for 2 h, and stored at −80°C for later analysis. Also, blood from six donors was prepared and incubated as described above with either no air in tubes, ambient air bubbles, or ambient air bubbles and either the mouse anti-human factor D mAb (clone 166-32; kindly provided by Genentech, South San Francisco, CA) in a final concentration of 50 μg/ml or Cp40 in a final concentration of 20 µM (three donors).

### Blood gas experiments

We obtained blood from six donors, and for each donor, we prepared one baseline tube, three tubes with no air, and three tubes with air bubbles as described above. We drew 1 ml blood from the baseline tube on a safePICO heparinized syringe (Radiometer, Copenhagen, Denmark) and analyzed the blood on an epoc MC55A blood analyzer (Siemens Healthcare GmbH, Eschborn, Germany), and incubated the no-air and air tubes at 37°C on a Rock’n’Roller tube roller mixer. After 60, 120, and 180 min of incubation, we removed a set of samples with and without air from the incubator, drew 1 ml of blood on a safePICO syringe, and analyzed the samples on the epoc MC55A.

### Antifoam experiments

In pilot experiments, we found the required amount of the pure silicone polymer antifoam A (Merck KGaA, Darmstadt, Germany) to be at least 1 μl/5 ml blood and antifoam B (10% polydimethylsiloxane in aqueous solution; Merck) to be at least 2.5 μl/5 ml blood for a full antifoaming effect during continuous bubbling of air through the blood. To examine for possible adverse effects of the antifoam reagents on the complement and coagulation systems, we collected blood from six donors, as described above. From each donor, we transferred 1 ml blood to three 1.8-ml Nunc tubes. We added either 2 μl antifoam A, 6 μl antifoam B, or nothing to these aliquots and incubated the samples for 180 min at 37°C on a Rock’n’Roller tube roller mixer. After incubation, we added EDTA, isolated plasma, and stored the samples for subsequent analysis as described above.

### Plasma experiments

We collected blood from six donors in Nunc polypropylene tubes prefilled with lepirudin to a final concentration of 50 µg/ml blood as described above. We centrifuged the tubes at 3000 *g* for 20 min at RT and transferred the supernatant to 2 ml Biopur polypropylene Eppendorf tubes (Eppendorf, Hamburg, Germany). We centrifuged these tubes at 10,000 *g* for 30 min at RT and transferred the cell-free plasma to 50 ml polypropylene conical Falcon tubes placed on a block heater at 37°C. We transferred aliquots of 358 μl plasma to 1.8-ml Nunc tubes prefilled with 142 µl sterile PBS with MgCl_2_ and CaCl_2_ and aliquots of 2506 μl plasma to 5-ml Nunc tubes prefilled with 994 μl PBS. From there, we transferred the diluted plasma to 1.8-ml Nunc tubes, filling the tubes and the tube lids completely. From three donors, we transferred 358-µl aliquots to 1.8-ml Nunc tubes containing 40 µM Cp40 or 200 µg/ml eculizumab in PBS to a final volume of 142 µl, yielding equipotent concentrations of inhibitors in blood and plasma experiments (assuming a plasma volume of 50%). After collecting baseline samples, we added air bubbles to all tubes, except no-air tubes, as described under *Whole blood experiments* above, and incubated the tubes for 180 min at 37°C on a Rock’n’Roller, added EDTA to a final concentration of 20 mM, transferred the plasma to 1-ml Matrix storage tubes, and stored these at −80°C for later analysis.

### Complement and hemostasis analyses

In EDTA plasma from whole blood and plasma incubations, we analyzed C3bc, C3bBbP, and the terminal complement complex sC5b9 (TCC) by ELISAs developed at our laboratory, described in detail previously (23, 27, 28), using pooled human sera from healthy donors activated with zymosan (10 mg/ml) and heat-aggregated human IgG (1 mg/ml) as a reference standard set to 1000 complement arbitrary units (CAU/ml). We analyzed C4d using the MicroVue C4d fragment enzyme immunoassay (EIA) kit (Quidel Corporation, Athens, OH), C4a using the MicroVue C4a fragment EIA kit (Quidel), C3a using the MicroVue C3a Plus EIA kit (Quidel), C5a using a kit from Hycult Biotech (Uden, the Netherlands), PTF1+2 using the Enzygnost F1+2 kit (Siemens Healthcare, Marburg, Germany), and BTG using the Human CXCL7/NAP-2 DuoSet ELISA kit (R&D Systems, Abingdon, UK). We analyzed MP-TF in citrated plasma using the Zymuphen MP-TF kit (Hyphen Biomed, Neuville de Oise, France). We conducted all analyses as per the manufacturers’ instructions. Due to slight changes in the protocol during the study period, C4d from 9, C3bBbP from 10 and BTG from 15 whole-blood incubations were analyzed.

### Cytokine analysis

In EDTA plasma from 13 whole-blood incubations, we analyzed the following cytokines using the Bio-Plex human 27-plex kit and the Bio-Plex 200 system (Bio-Rad Laboratories, Hercules, CA): IL-1β, IL-1 receptor antagonist, IL-2, IL-4, IL-5, IL-6, IL-7, IL-8, IL-9, IL-10, IL-12, IL-13, IL-15, IL-17, MCP-1, MIP-1α, MIP-1β, eotaxin-1, IP-10, basic fibroblast growth factor (bFGF)6, G-CSF, GM-CSF, IFN-γ, platelet-derived growth factor-BB (PDGF-BB), RANTES, TNF, and vascular endothelial growth factor.

### Gene expression analysis

All equipment used was RNase- and DNase-free. From thawed, PAXgene-treated whole blood, two aliquots of 1.25 ml were transferred to 2-ml Eppendorf tubes, and the tubes were centrifuged at 5000 *g* for 10 min. The supernatant was carefully poured from each tube, and the pellets were resuspended in 0.2 ml PBS without calcium or magnesium. From the resuspended cell lysate, we isolated total RNA using the MagNA Pure 96 Instrument and MagNA Pure 96 Cellular Large Volume Kit (Roche Diagnostics GmbH, Mannheim, Germany) as per the manufacturer’s instructions. We analyzed the RNA concentrations using a Thermo Scientific NanoDrop spectrophotometer (Life Technologies, Carlsbad, CA) and RNA integrity number using an Agilent 2100 Bioanalyzer (Agilent, Santa Clara, CA). The mean RNA integrity number was 8.0. We used the TaqMan RNA-to-Ct 1-Step Kit (Thermo Fisher Scientific) for gene expression studies as per the manufacturer’s instructions. We used 20 ng RNA in a total reaction volume of 20 µl. Cycling conditions were set according to the kit insert, and qPCRs were run in triplicates in MicroAmp Fast 96-Well Reaction Plates on the QuantStudio 6 Real-time PCR System instrument (Thermo Fisher Scientific) using predeveloped TaqMan gene expressions assays for the candidate gene tissue factor (TF, Hs01076029_m1, F3; Thermo Fisher Scientific) and the human β_2_-microglobulin (B2M, 4333766F; Thermo Fisher Scientific) as an endogenous control. The relative tissue factor mRNA (TF-mRNA) levels were quantified using the comparative ΔΔCt method. All results are given as fold changes (relative quantification) compared with blood incubated without air.

### Statistical methods

Data were organized and fold change was calculated in Excel for Mac 16.37 (Microsoft Corporation, Redmond, WA). Prism 8.4.2 for Mac (GraphPad Software, La Jolla, CA) was used for statistical analysis and graphing, except for cytokine imputations and statistics. These were calculated in STATA for Windows version 16 (StataCorp LLC, College Station, TX). Before statistical analysis, we logarithmically transformed all complement activation product readouts and PTF1+2, BTG, and cytokine readouts to fit a Gaussian distribution. To avoid missing values due to results missing at random, we excluded two donors from the complement and PTF1+2 datasets by listwise deletion; one sample incubated with Cp40 was lost, and one donor had an extreme C3bc value outlier. The complete datasets were analyzed using a repeated-measures ANOVA with Greenhouse-Geisser correction to account for the within-subject correlations. The effect of anti-factor D and Cp40 on C3bc and C3bBbP was analyzed using a mixed model due to the incomplete data. Due to corrupted measurements of cytokines in three incubations with air bubbles and eculizumab, three with air and PMX53, and four with air bubbles and combined r18D11 and Cp40, we applied multiple imputations by chained equations under the missing at random assumption. We analyzed the data using a linear mixed-effects model with random intercept. The imputation of missing cytokine values was performed to correct the bias in estimates otherwise emerging from the high between-subject compared with within-subject variability in the data. Blood gas readouts from each sample time point were compared using multiple *t* tests. All *p* values were corrected for multiple testing by the Benjamini-Hochberg method for controlling the false discovery rate. We considered a *p* value <0.05 significant for all analyses. Data are presented as means with 95% confidence intervals (95% CI).

## Results

### Whole-blood experiments

#### Complement activation

**FIGURE 2.**
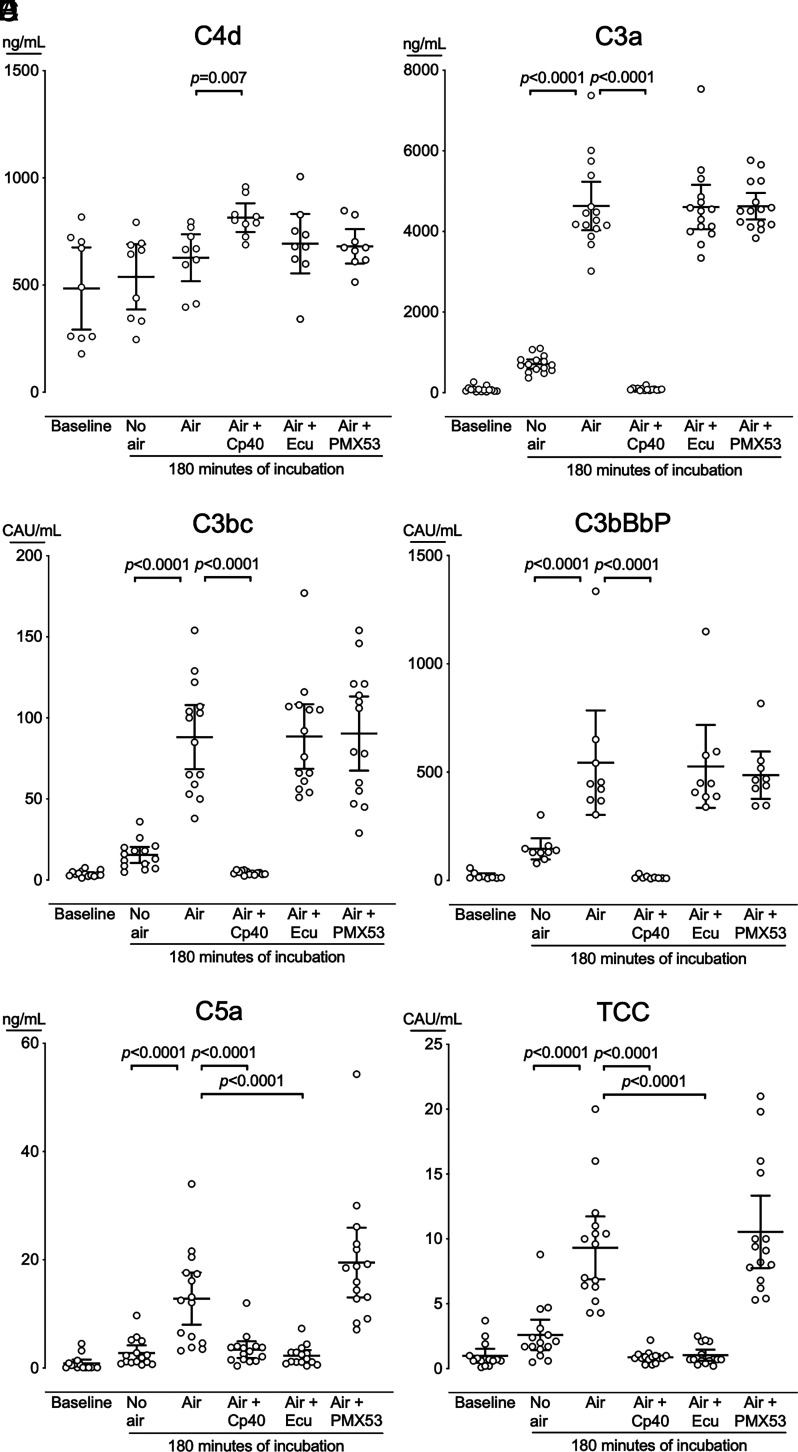
Complement activation in blood incubated without or with air bubbles. Human whole blood from 16 donors was incubated for 180 min in either completely blood-filled tubes (no air); with air bubbles in the tubes (air); or air bubbles and the C3 inhibitor Cp40, the C5 inhibitor eculizumab (Ecu), or the C5a receptor 1 inhibitor PMX53. The blood was analyzed for C4d (**A**), C3a (**B**), C3bc (**C**), C3bBbP (**D**), C5a (**E**), and TCC (**F**). Horizontal lines represent means. Error bars are 95% confidence intervals. Only significant *p* values (*p* < 0.05) are shown.

C4d at baseline was 483 ng/ml (95% CI, 292 to 678 ng/ml) (Figure 2A). After incubation, C4d was 538 ng/ml (95% CI, 386 to 690 ng/ml), in blood incubated without air compared with 628 ng/ml (95% CI, 518 to 737 ng/ml), in blood incubated with air bubbles (*p* = 0.09). C3a at baseline was 80 ng/ml (95% CI, 43 to 117 ng/ml) (Figure 2B). After incubation, C3a was 709 ng/ml (95% CI, 593 to 825 ng/ml), in blood incubated without air compared with 4632 ng/ml (95% CI, 4031 to 5233 ng/ml), in blood incubated with air bubbles (*p* < 0.0001). C3bc at baseline was 3.8 CAU/ml (95% CI, 2.8 to 4.8 CAU/ml) (Figure 2C). After incubation, C3bc was 16 CAU/ml (95% CI, 11 to 20 CAU/ml), in blood incubated without air compared with 88 CAU/ml (95% CI, 68 to 108 CAU/ml), in blood incubated with air bubbles (*p* < 0.0001). C3bBbP at baseline was 21 CAU/ml (95% CI, 8.3 to 33 CAU/ml) (Figure 2D). After incubation, C3bBbP was 146 CAU/ml (95% CI, 97 to 195 CAU/ml), in blood incubated without air compared with 544 CAU/ml (95% CI, 303 to 785 CAU/ml), in blood incubated with air bubbles (*p* < 0.0001).

C5a at baseline was 0.9 ng/ml (95% CI, 0.1 to 1.6 ng/ml) (Figure 2E). After incubation, C5a was 2.8 ng/ml (95% CI, 1.4 to 4.2 ng/ml), in blood incubated without air compared with 13 ng/ml (95% CI, 8.0 to 18 ng/ml) in blood incubated with air bubbles (*p* < 0.0001). TCC at baseline was 1.0 CAU/ml (95% CI, 0.5 to 1.5 CAU/ml) (Figure 2F). After incubation TCC was 2.6 CAU/ml (95% CI, 1.5 to 3.8 CAU/ml), in blood incubated without air compared with 9.3 CAU/ml (95% CI, 6.9 to 12 CAU/ml), in blood incubated with air bubbles (*p* < 0.0001).

#### Complement inhibitors and correlations: early (C3) versus late (TCC) activation

In blood incubated with air bubbles and the C3 inhibitor Cp40, C3a, C3bc, C3bBbP, C5a, and TCC were reduced to baseline values (*p* < 0.0001) (Figure 2B–F). In incubations with air bubbles and the C5 inhibitor eculizumab, C5a and TCC were reduced to baseline values (*p* < 0.0001) (Figure 2E and F). In blood incubated with air bubbles, C4d was not inhibited by eculizumab, but C4d was increased by Cp40 (*p* = 0.007) (Figure 2A). As expected, the C5aR1 antagonist PMX53 did not reduce the complement activation products (Figure 2A–F).

**FIGURE 3.**
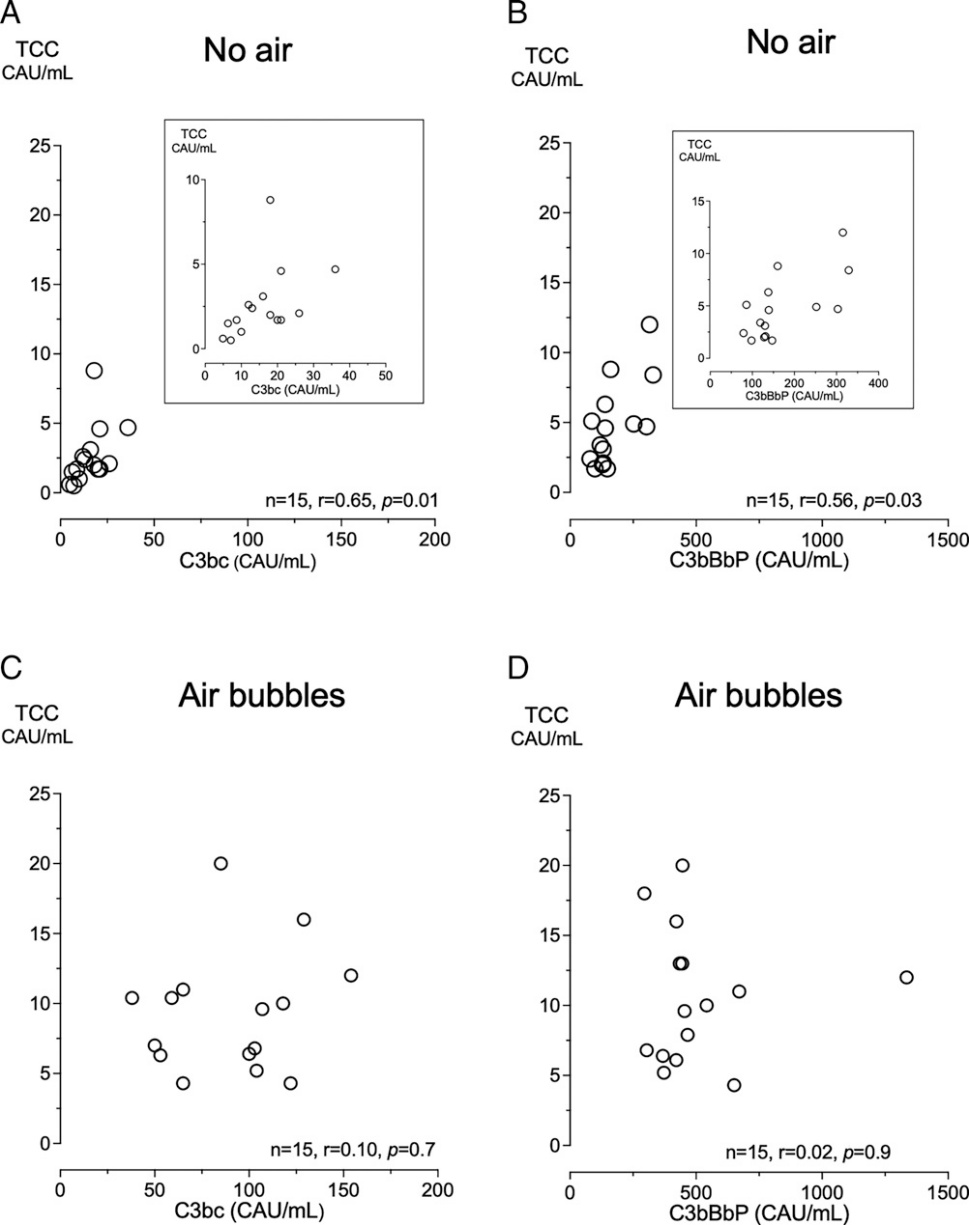
Correlation between early (C3bc and C3bBbP) and late (TCC) complement activation. After 180 min of incubation of human whole blood, there was a strong Spearman correlation between C3bc and TCC and a moderate correlation between C3bBbP and TCC in samples incubated without air (**A** and **B**), but there was no correlation in samples incubated with air bubbles (**C** and **D**).

In blood incubated without air, an increase in alternative and terminal pathway activation was found, with a strong correlation between C3bc and TCC (*r* = 0.65; *p* = 0.01) (Figure 3A) and a moderate correlation between C3bBbP and TCC (*r* = 0.56; *p* = 0.03) (Figure 3B). In blood incubated with air bubbles, the relative increase in alternative pathway activation was substantially higher than the terminal pathway, and no correlations were seen between C3bc and TCC (*r* = 0.10; *p* = 0.7) (Figure 3C) or C3bBbP and TCC (*r* = 0.02; *p* = 0.9) (Figure 3D).

#### Complement alternative pathway activation: anti-factor D versus Cp40

**FIGURE 4.**
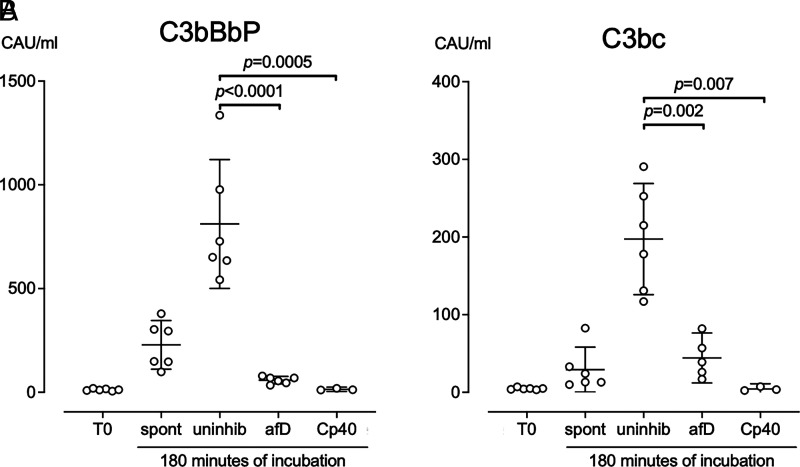
The specific role of the alternative pathway. Lepirudin-anticoagulated human whole blood from six donors (Cp40 was incubated in blood from three donors) was incubated for 180 min on a roller mixed at 37°C with no air in tubes (spont), with ambient air bubbles (uninhib), or with ambient air bubbles and either anti-factor D (afD) to a final concentration of 50 μg/ml or the C3 blocking peptide Cp40 to a final concentration 20 µM. Plasma was analyzed for C3bc (**A**) and C3bBbP (**B**) using ELISA. Horizontal lines represent means. Error bars are 95% confidence intervals.

To precisely delineate the role of the alternative pathway compared with the classical and lectin pathways in air bubble–induced C3 activation, C3BbP and C3bc formation was evaluated in the absence versus presence of a specific anti-human factor D Ab or the C3 inhibitor Cp40. After incubation of blood with air bubbles and no inhibitor, C3bBbP levels were 811 CAU/ml (95% CI, 501 to 1122 CAU/ml), compared with 59 CAU/ml (95% CI, 41 to 76 CAU/ml), in incubations with anti-factor D (*p* < 0.0001) and 14 CAU/ml (95% CI, 3.2 to 25 CAU/ml), in incubations with Cp40 (*p* = 0.0005) (Figure 4A). In contrast, after incubation of blood with air bubbles and no inhibitor, C3bc was only increased to 197 CAU/ml (95% CI, 126 to 269 CAU/ml), underscoring the importance of the alternative pathway in air bubble–induced C3 activation (Fig 4B). Compared with incubations without inhibitor, C3bc levels were reduced to 44 CAU/ml (95% CI, 12 to 77 CAU/ml), in incubations with anti-factor D (*p* = 0.002) and nearly abolished with a reduction to 4.4 CAU/ml (95% CI, 0 to 11 CAU/ml), in incubations with Cp40 (*p* = 0.007). This observation indicates a minor but genuine contribution of the classical pathway and the lectin pathway to air bubble–induced C3 activation, which was also reflected as a slight accumulation of C4d in the presence of Cp40 (Figure 2A).

#### Hemostasis and effect of complement inhibition

**FIGURE 5.**
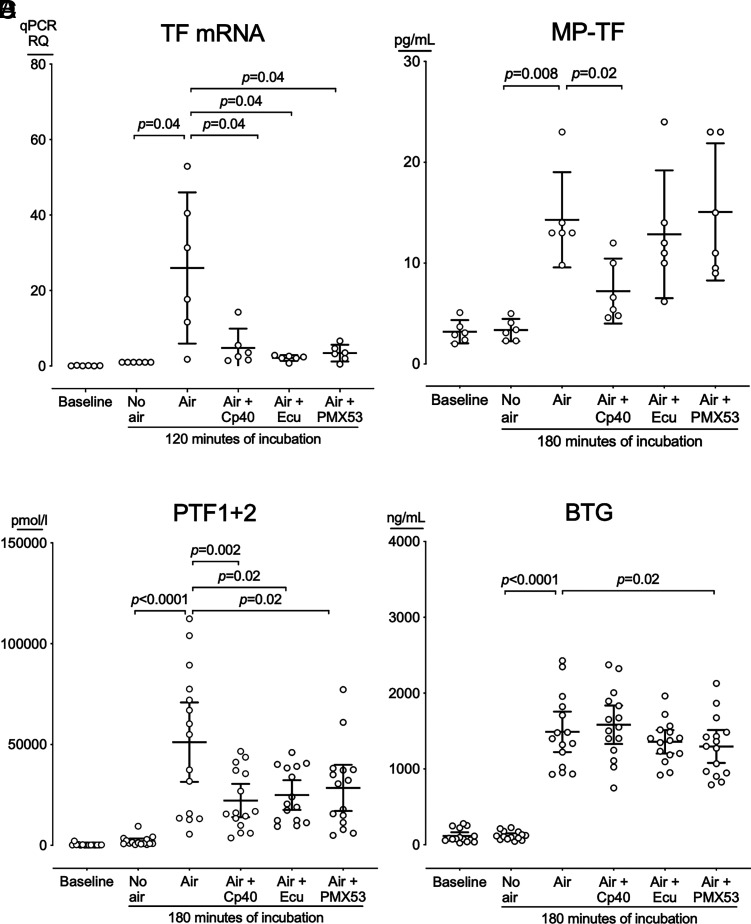
Hemostasis activation in blood incubated without or with air bubbles. Human whole blood from six donors was incubated with either no air; air bubbles (air); or air bubbles and Cp40, eculizumab, or PMX53. TF-mRNA was analyzed after 120 min (**A**), MP-TF (**B**), PTF1 + 2 (**C**), and BTG (**D**) were analyzed after 180 min. Horizontal lines represent means. Error bars are 95% confidence intervals. Only significant *p* values (*p* < 0.05) are shown.

TF-mRNA comparative ΔΔCt (relative quantification) increased 26-fold in blood incubated with air bubbles compared with blood incubated without air (*p* = 0.04) (Figure 5A). The three complement inhibitors substantially reduced TF-mRNA virtually to baseline: Cp40 by 5.4-fold (*p* = 0.04), eculizumab by 12-fold (*p* = 0.04), and PMX53 by 7.6-fold (*p* = 0.04).

MP-TF at baseline was 3.2 pg/ml (95% CI, 2.0 to 4.4 pg/ml) (Figure 5B). After incubation without air, MP-TF was 3.4 pg/ml (95% CI, 2.3 to 4.5 pg/ml), compared with 14 pg/ml (95% CI, 9.6 to 19 pg/ml), in incubations with air bubbles (*p* = 0.008). MP-TF was reduced by Cp40 to 7.2 pg/ml (95% CI, 4.0 to 10 pg/ml) (*p* = 0.02) but was not reduced by eculizumab or PMX53.

PTF1+2 at baseline was 336 pmol/l (95% CI, 54 to 618 pmol/l) (Figure 5C). After incubation without air, PTF1+2 was 2071 pmol/l (95% CI, 761 to 3381 pmol/l), compared with 51,166 pmol/l (95% CI, 31,468 to 70,863 pmol/l), in incubations with air bubbles (*p* < 0.0001). The three complement inhibitors substantially reduced PTF1+2: Cp40 to 22,222 pmol/l (95% CI, 13,974 to 30,471 pmol/l) (*p* = 0.002), eculizumab to 24,950 pmol/l (95% CI, 17,582 to 32,318 pmol/l) (*p* = 0.02), and PMX53 to 28,499 pmol/l (95% CI, 17,043 to 39,956 pmol/l) (*p* = 0.02).

BTG at baseline was 117 ng/ml (95% CI, 69 to 166 ng/ml) (Figure 5D). After incubation without air, BTG was 121 ng/ml (95% CI, 91 to 151 ng/ml), compared with 1489 ng/ml (95% CI, 1222 to 1756 ng/ml), in blood incubated with air bubbles (*p* < 0.0001). PMX53 reduced BTG to 1297 ng/ml (95% CI, 1080 to 1513 ng/ml) (*p* = 0.02), whereas Cp40 or eculizumab did not reduce BTG significantly.

#### Cytokine release and effect of complement inhibition

**FIGURE 6.**
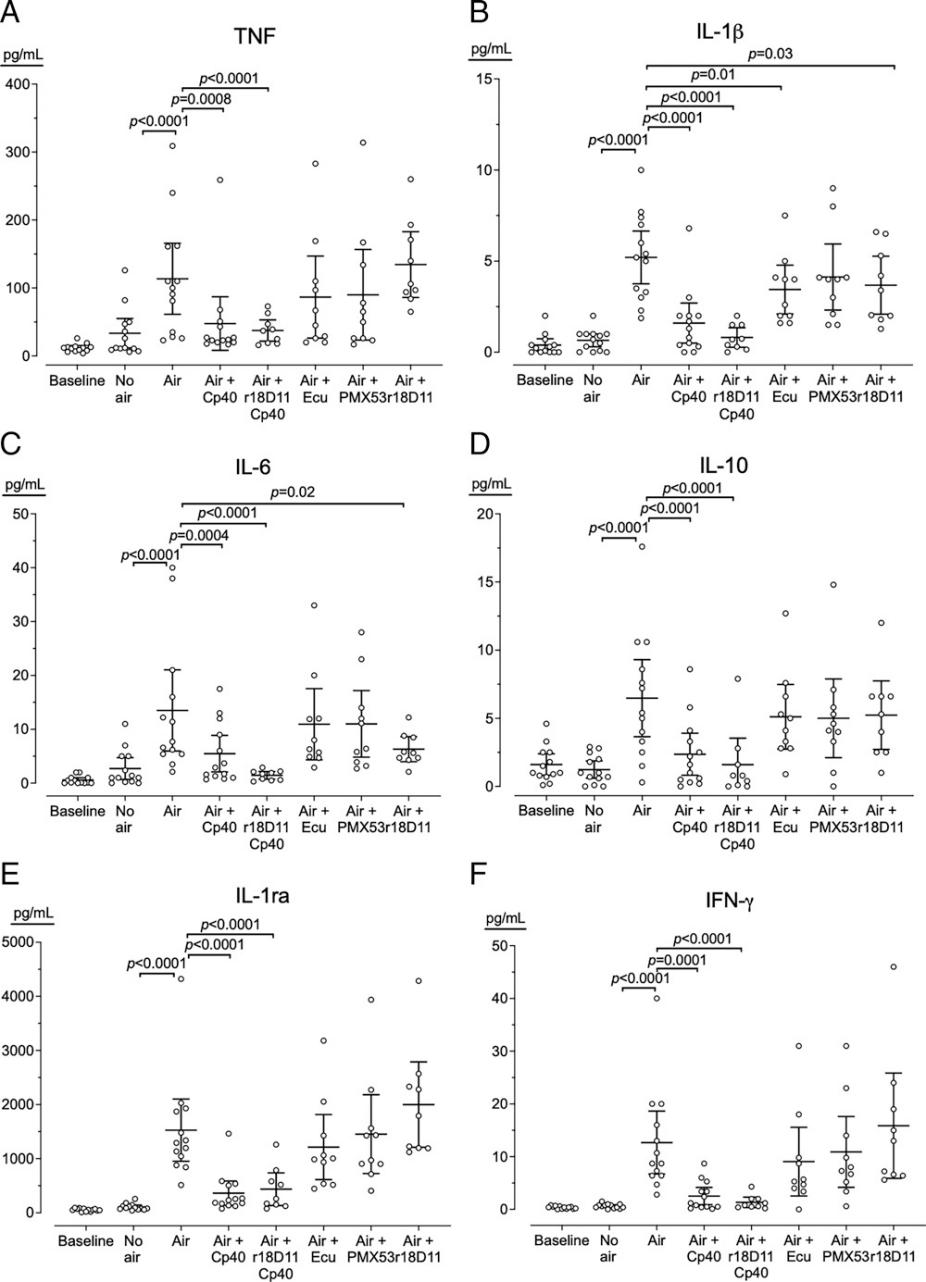
Pro- and anti-inflammatory cytokine released in blood incubated without or with air bubbles. Human whole blood from 13 donors was incubated 180 min with either no air; air bubbles (air); or air bubbles and Cp40, Cp40 and r18D11 (aCD14), eculizumab (Ecu), PMX53, or r18D11. The blood was analyzed for the cytokines TNF (**A**), IL-1β (**B**), IL-6 (**C**), IL-10 (**D**), IL-1 receptor antagonist (IL-1ra) (**E**), and IFN-γ (**F**). Horizontal lines represent means. Error bars are 95% confidence intervals. Only significant *p* values (*p* < 0.05) are shown.

**FIGURE 7.**
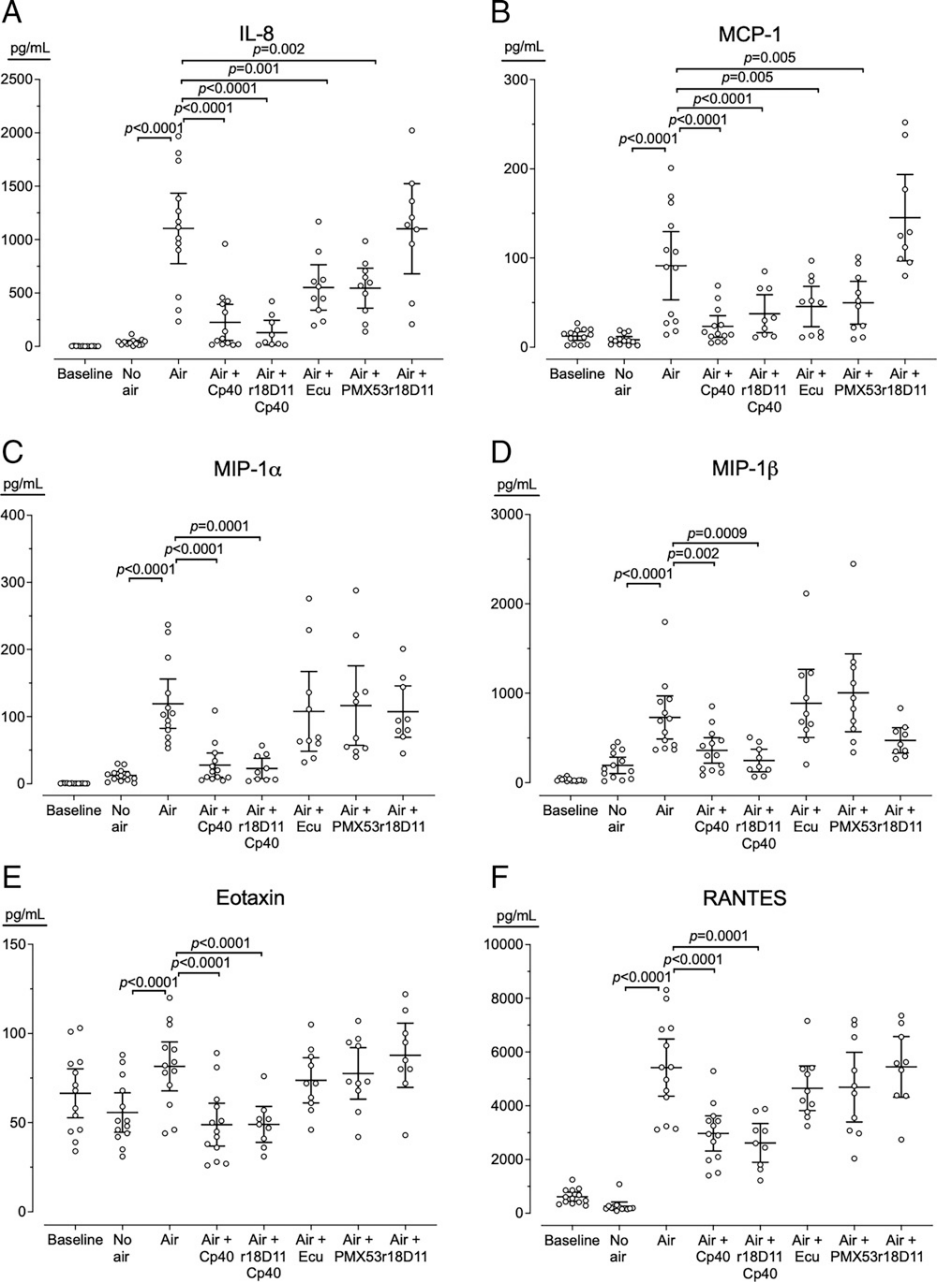
Chemokines released in blood incubated without or with air bubbles. Human whole blood from 13 donors was incubated 180 min with either no air; air bubbles (air); or air bubbles and Cp40, Cp40 and r18D11 (aCD14), eculizumab (Ecu), PMX53, or r18D11. The blood was analyzed for the chemokines IL-8 (**A**) MCP-1 (**B**), MIP-1α (**C**), MIP-1β (**D**), eotaxin (**E**), and RANTES (**F**). Horizontal lines represent means. Error bars are 95% confidence intervals. Only significant *p* values (*p* < 0.05) are shown.

Whole blood incubated with air bubbles induced, on average, an 11-fold increase (range, 1.5–78) in 25 of 27 cytokines (*p* < 0.0001) (Table I), including the classical pro- and anti-inflammatory cytokines (Figure 6A–F) and chemokines (Figure 7A–F). Because cytokines are frequently induced both by complement and by the TLRs, the inhibition of the TLR coreceptor CD14 by anti-CD14 Ab r18D11 was included. Cp40 reduced all the 25 cytokines, on average, 2.7-fold (range, 1.4–4.9) (*p* < 0.05) (Table I). Cp40 and r18D11 combined reduced all the 25 cytokines 3.9-fold (range, 1.3–9.5) (*p* < 0.003). r18D11 alone reduced only IL-1β (Figure 6B), IL-6 (Figure 6C), and bFGF, on average, 6-fold (*p* < 0.04) (Table I). Inhibition at the level of C5 with eculizumab reduced only IL-1β (Figure 6B), IL-8 (Figure 7A), and MCP-1 (Figure 7B), on average, 1.7-fold (*p* < 0.03), and inhibition of C5aR1 with PMX53 reduced only IL-8 (Figure 7A) and MCP-1 (Figure 7B), on average, 1.9-fold (*p* < 0.006) (Table I).

**Table I.** Cytokine response to incubation without or with air bubbles or with air and inhibitors of either C3, C3 and CD14 combined, C5, or C5a receptor 1

Cytokine	No Air Versus Air Bubbles		Air Bubbles Versus Air Bubbles and Cp40		Air Bubbles Versus Air Bubbles and Cp40/r18D11		Air Bubbles Versus Air Bubbles and Eculizumab		Air Bubbles versus Air Bubbles and PMX53	
	Fold Increase	FDR Adjusted *p* Value^*a*^	Fold Decrease	FDR Adjusted *p* Value*^a^*	Fold Decrease	FDR Adjusted *p* Value*^a^*	Fold Decrease	FDR Adjusted *p* Value*^a^*	Fold Decrease	FDR Adjusted *p* Value*^a^*
PDGF-BB	78	**<*0.0001***	2.9	**<*0.0001***	5.6	**<*0.0001***	1.1	0.5	1	0.7
IL-8	29	**<*0.0001***	4.9	**<*0.0001***	8.6	**<*0.0001***	2	***0.001***	2	***0.002***
IFN-γ	20	**<*0.0001***	5	***0.0001***	9.5	**<*0.0001***	1.4	0.2	1.2	0.4
RANTES	20	**<*0.0001***	1.8	**<*0.0001***	2.1	***0.0001***	1.2	0.2	1.2	0.3
IL-1ra	14	**<*0.0001***	4.2	**<*0.0001***	3.5	**<*0.0001***	1.3	*0.3*	1.1	0.5
G-CSF	14	**<*0.0001***	3.3	***0.0005***	3.4	***0.0004***	1	0.7	0.9	0.7
VEGF	11	**<*0.0001***	4.2	**<*0.0001***	4.4	**<*0.0001***	1.2	0.4	1.1	0.4
MCP-1	11	**<*0.0001***	3.9	**<*0.0001***	2.4	**<*0.0001***	2	***0.005***	1.8	***0.005***
MIP-1α	9.6	**<*0.0001***	4.3	**<*0.0001***	5.2	***0.0001***	1.1	0.4	1	0.6
IL-1β*^b^*	8	**<*0.0001***	3.2	**<*0.0001***	6.4	**<*0.0001***	1.5	***0.02***	1.3	0.2
IL-17α	7.2	**<*0.0001***	2.1	**<*0.0001***	2.8	**<*0.0001***	1.1	0.4	1.1	0.3
IL-15	5.8	**<*0.0001***	2.1	***0.0004***	3.6	**<*0.0001***	1	0.8	1.1	0.5
IL-10	5.2	**<*0.0001***	2.7	**<*0.0001***	4	**<*0.0001***	1.3	0.2	1.3	0.2
IL-6*^b^*	5	**<*0.0001***	2.5	***0.0004***	9.3	**<*0.0001***	1.2	0.2	1.2	0.2
GM-CSF	4.9	**<*0.0001***	1.5	***0.04***	4.4	***0.0001***	0.9	0.8	1	0.7
IL-9	4.7	**<*0.0001***	1.4	***0.02***	1.3	***0.002***	1	0.5	1	0.4
IL-5^*c*^	4.3	**<*0.0001***	2.9	***0.0001***	3	***0.0001***	1	0.7	1	0.8
bFGF*^b^*	4.1	**<*0.0001***	1.5	***0.0008***	2.4	**<*0.0001***	1.1	0.3	1.1	0.4
IL-2	4	**<*0.0001***	1.9	***0.0001***	3.4	***0.0001***	1.1	0.3	1.2	0.3
MIP-1β	3.8	**<*0.0001***	2	***0.002***	3	***0.0009***	0.8	0.8	0.7	*0.4*
IL-4	3.5	**<*0.0001***	2.2	***0.0005***	1.9	***0.0002***	1.1	0.4	1.1	0.5
TNF	3.4	**<*0.0001***	2.4	***0.0008***	3	**<*0.0001***	1.3	0.3	1.3	0.3
IL-7	2.3	**<*0.0001***	1.7	***0.003***	1.6	***0.0002***	1.2	0.1	1	0.3
IL-13	1.8	**<*0.0001***	1.5	***0.0003***	1.5	***0.0002***	1.1	0.3	1	0.4
Eotaxin	1.5	**<*0.0001***	1.7	**<*0.0001***	1.7	**<*0.0001***	1.1	0.2	1.1	0.4

a Log-transformed data. Linear mixed-effects model with random intercept. Multiple comparisons corrected using the FDR method with *q* = 0.05. Discoveries (FDR adjusted *p* < 0.05) are shown in italic and bold type.

b Compared with incubations with air bubbles only, incubations with air bubbles and r18D11 reduced IL-1β 6.4-fold (*p* = 0.03^a^), IL-6 9.3-fold (*p* = 0.02^a^), and bFGF 2.4-fold (*p* = 0.05^a^).

c Due to low bead number, only nine incubations were analyzed for IL-5.

FDR, false discovery rate; IL-1ra, IL-1 receptor antagonist; VEGF, vascular endothelial growth factor.

#### Blood gas and chemistry

In blood incubated with air bubbles compared with blood incubated without air, pO_2_, pCO_2_, pH, lactate, ionized Ca^2+^, and HCO_3_^−^ did not differ at the start of the incubation (Supplemental Fig. 2A–F). Throughout the incubation period, pO_2_ (Supplemental Fig. 2A), lactate (Supplemental Fig. 2B), and pH (Supplemental Fig. 2C) were higher and pCO_2_ (Supplemental Fig. 2D) was lower in blood incubated with air bubbles than in blood incubated without air (*p* < 0.0001, *p* < 0.03, *p* < 0.05, and *p* < 0.003, respectively). At the end of the 180-min incubation, ionized Ca^2+^ (Supplemental Fig. 2E) and HCO_3_^−^ (Supplemental Fig. 2F) were lower in blood incubated with air bubbles (*p* = 0.03 and *p* = 0.01, respectively).

#### Effects of antifoam on complement and coagulation

Possible adverse effects of antifoam were examined in blood incubated with antifoam A or B. Antifoam B substantially increased C3bc, TCC, and PTF1+2 (*p* < 0.0001, *p* < 0.0001, and *p* = 0.0002, respectively) compared with blood incubated without antifoam (Supplemental Fig. 3A–C). Antifoam A did not affect these readouts. In our experimental setup, we avoided foam formation by incubating tubes with blood and air bubbles on a roller mixer rather than continuously bubbling air through the blood. This eliminated the need for the addition of antifoam agents that could induce adverse effects in the blood.

### Plasma experiments

C4d did not differ between plasma incubated without air or with air bubbles (Supplemental Fig. 4A). C3bc was 35 CAU/ml (95% CI, 17 to 54 CAU/ml) in plasma incubated without air compared with 157 CAU/ml (95% CI, 120 to 193 CAU/ml) in plasma incubated with air bubbles (*p* = 0.004) (Supplemental Fig. 4B). C3bBbP was 296 CAU/ml (95% CI, 183 to 408 CAU/ml) in plasma incubated without air compared with 1219 CAU/ml (95% CI, 783 to 1654 CAU/ml) in plasma incubated with air bubbles (*p* = 0.004) (Supplemental Fig. 4C). TCC was 5.3 CAU/ml (95% CI, 0 to 12 CAU/ml) in plasma incubated without air compared with 18 CAU/ml (95% CI, 2.0 to 34 CAU/ml) in plasma incubated with air bubbles, but the difference was nonsignificant (*p* = 0.09) (Supplemental Fig. 4D). Notably, in contrast to whole blood, PTF1+2 did not differ between incubations with air bubbles and without air (Supplemental Fig. 4E). As expected, the C3 inhibitor Cp40 abolished the C3bc and C3bBbP formation, and both Cp40 and eculizumab abolished the TCC formation (Supplemental Fig. 4B–D).

## Discussion

In the present study, we used air bubbles incubated in a whole-blood model of venous air emboli. We showed how air bubbles induced substantial thromboinflammation, including activation of complement, coagulation, and platelets, and the release of 25 cytokines, including ILs, chemokines, and growth factors. This inflammatory response was mainly C3 driven, and C3 inhibition substantially reduced hemostasis, measured by TF-mRNA, MP-TF, and PTF1+2 and all 25 cytokines.

In contrast, during air bubbling, C5 activation was less pronounced and showed no correlation with C3 activation. C5 inhibition reduced TF-mRNA and PTF1+2 to the same extent as C3 inhibition and marginally inhibited platelet BTG release, which was the only marker that C3 did not inhibit. Interestingly, C5 inhibition reduced only three cytokines, IL-1β, IL-8, and MCP1, and the effect of C5 inhibition was mainly C5aR1 mediated. Surprisingly, inhibiting only CD14, an important cofactor for the TLRs typically inducing cytokines in blood incubated with bacteria (29, 30), reduced only IL-1β, IL-6, and bFGF. In contrast, C3 inhibition reduced all 25 cytokines, and combined C3 and CD14 inhibition further potentiated the reduction. To our knowledge, this study is the first human whole-blood study elucidating the role of complement in air bubble–induced hemostasis and cytokine release, revealing a crucial role for C3 in thromboinflammation. The alternative pathway was the primary driver of this C3 activation because blocking of factor D and C3 equally reduced the levels of C3bc and C3bBbP.

Oxygen, nitrogen, and air bubbles have previously been shown in vitro in serum and blood to change the C3 configuration to iC3, also termed “C3b-like” or “C3(H_2_O)” (10), indicating that the complement activation depends on the gas–plasma interface on the surface of the air bubbles per se more than on the composition of gas inside the bubbles. This is in accordance with the hypothesis put forward by Atkinson and Farries that complement can discriminate between a “foreign surface” and a “self-surface” through the binding of the alternative pathway regulatory proteins, factors B and H (31); C3 binds the activating factor B with a higher affinity than the inhibiting factor H on a foreign surface. Thus, it is reasonable to suggest that the blood–gas surface interaction acts at a foreign surface and triggers activation.

A pivotal step in the complement activation by air bubbles is the hydrolysis and conformational change of the C3 molecule to C3(H_2_O), as described several decades ago (10, 32). In line with these studies, we found that air bubbles triggered a strong generation of the C3 convertase C3bBbP, suggesting a similar activation mechanism. Notably, in blood incubated with air bubbles, there was no correlation between C3 activation (C3bBbP or C3bc) and C5 activation (TCC). We have introduced the term “dissociation” of complement to indicate the different activation potency of the convertases, with a highly efficient C3 convertase and a C5 convertase with low or no activity. This rarely seen but well-known phenomenon is discussed below.

Complement activation frequently occurs on particles or solid surfaces such as microbes or damaged endothelium, and typically an efficient C5 convertase is generated. The potent mediator C5a induces a strong inflammatory response through its receptor C5aR1 and, to a lesser extent, through C5aR2 (33). An example of this is described in a recent study of patients with coronavirus disease 2019 (COVID-19) (34), in whom complement activation products from the classical (C4d), the alternative (C3bBbP), the common (C3bc), and the terminal pathway (C5a and sC5b-9) were measured. In that study, patients with COVID-19 had a typical “sepsis-like” complement system activation, with a significant and strong correlation between all activation products. Under such conditions, inhibition of C5, C5a, or C5aR1 dampens the inflammatory response generated from the terminal pathway, and, although approved for use only for a couple of rare diseases (35), an inhibitor of C5 cleavage is the only complement inhibitor in routine clinical use. Interestingly, C3 inhibition in patients with COVID-19 afforded broader therapeutic control by attenuating both C3a and sC5b-9 generation and preventing factor B consumption, which is associated with a more robust decline of neutrophil counts, attenuated neutrophil extracellular trap release, faster serum lactate dehydrogenase decline, and more prominent lymphocyte recovery (36).

In contrast to this “whole complement system activation,” which involves the two central components, C3 in the early phase and C5 in the terminal phase, we have previously described a “dissociation” phenomenon between C3 and C5 activation in patients with C3 nephritic factors (C3NeFs) (37). NeFs are autoantibodies binding to and stabilizing the complement convertases. In patients with C3NeF, the C3 convertase continuously activates C3, whereas the C5 convertase is inefficiently formed with minimal or no terminal pathway activation. The “dissociation” phenomenon has been confirmed by others (38), and the NeFs are now classified as C4NeF, C3NeF, and C5NeF.

In the present study on air bubbles, the inflammatory response was mainly dependent on C3 and could hardly be attenuated by C5 inhibition. A few of the hemostatic readouts, including TF and PTF1+2, were dependent on C5a but equally well abolished by C3 inhibition, suggesting that air bubble–induced inflammatory responses should be inhibited at the level of C3. The mechanisms need to be further investigated, but the main limitation for further investigation of the role of C3 is the lack of a highly specific C3aR antagonist, because those currently available are not selective and have both agonistic and antagonistic effects (39). The differences observed in the effect of the C3 inhibitor Cp40 and C5 inhibitor eculizumab or the C5aR1 blocker PMX53 on the readouts TF-mRNA, MP-TF, BTG, and PTF1+2 could not be explained by insufficient amounts of inhibitor. On the basis of our experience using these inhibitors for more than a decade, we used all inhibitors well in excess of the required concentration. Furthermore, in pilot experiments in our model system, we increased the inhibitor concentration 10-fold with no additional inhibitory effect observed on MP-TF, BTG, or PTF1+2.

Bubbles triggered a statistically nonsignificant increase in C4d, in line with our previous findings (22). C3 inhibition further amplified the C4d increase. This indicates that air bubbles activated not only the alternative but also the classical or lectin pathways. The exact mechanisms for these findings have not been elucidated before, to our knowledge. However, we suggest some theoretical explanations. Bubbles might have activated the C4 directly by hydrolysis, such as the C3 activation (10), because both the C4 and C3 molecules contain internal thioester bonds, and activated C4 not covalently bound to a surface will rapidly be inactivated by hydrolyzation (40). Additionally, the C4 could also have been indirectly activated, for example, by cross-talk with activated platelets (20). Finally, C3 blocking could have led to an increased C4 activation with the release of C4d after C4b deposition to amino and hydroxyl groups normally occupied by activated C3, similar to complement deposition on bacteria, as we have observed (41). Whether a similar mechanism exists on the gas–blood interface is speculative and has not been documented but cannot be excluded.

Air bubbles triggered a vigorous complement-mediated cytokine response. Cp40 effectively attenuated the response for all cytokines, and the combination of Cp40 and r18D11 further reduced the response slightly. In contrast, single inhibition with eculizumab reduced only IL-1β, IL-8, and MCP1, implying that the modest activation taking place through the C5 convertase was still sufficient to induce these key cytokines, of which IL-8 previously has been shown to be mainly dependent on complement activation (42). Single inhibition with r18D11 reduced only IL-1β, IL-6, and bFGF. IL-1β is a major product following the formation of the inflammasomes, such as NLRP3 (43). Despite these interesting effects of C5 inhibition, collectively, our findings suggest that air bubbles activated inflammatory cells primarily through a C3-dependent mechanism.

Air bubbles activated the hemostasis with a substantial increase in the coagulation split product PTF1+2; the platelet-specific BTG; the cytokines RANTES and PDGF-BB, which are both released from activated platelets (44); and tissue factor, which can be released in microparticles from various cells, including monocytes (45). In contrast, in blood incubated without air, all readouts remained at baseline, showing that contact activation in the plastic tubes was not a major contributor to the hemostasis. In blood incubated with air bubbles, Cp40, eculizumab, and PMX53 abrogated TF-mRNA and equally reduced PTF1+2. The effects of eculizumab on air-induced TF-mRNA and PTF1+2 align with our observations in studies of *Escherichia. coli* in human whole blood (46). Cp40, but not eculizumab or PMX53, partly reduced MP-TF, RANTES, and PDGF-BB, suggesting both a C3-driven and a non–complement-driven TF release of presynthesized encrypted or intracellular TF in MP-TF from various blood cells, including monocytes (47). In contrast, de novo TF production, measured as an increase in TF-mRNA, was completely C5aR1 dependent. The BTG release from platelets was not reduced substantially by either Cp40, eculizumab, or PMX53, suggesting that the platelet activation was mainly non–complement dependent. The coagulation measured as PTF1+2 was activated both through a C5/C5aR1-dependent mechanism, possibly TF upregulation on the surface of monocytes, and through a complement-independent mechanism. PTF1+2 remained at baseline levels in plasma experiments, showing that the air-induced coagulation was highly dependent on blood cells. Both monocytes and platelets can activate coagulation (48), and we speculate that these cells played a pivotal role in the air-induced coagulation. However, our study was not designed to pinpoint the exact mechanisms for the hemostasis, and further studies are needed to elaborate on this in detail.

Importantly, our findings consistently support the use of C3 inhibition to reduce air-induced coagulation.

The results presented in this paper are based on the use of lepirudin anticoagulated whole blood without antifoam or ambient air in the tubes as an experimental system, which we suggest should be used to study thromboinflammation (23). In contrast to serum- and plasma-based models, whole blood contains both plasma proteins and all cell populations including platelets and leukocytes, as well as platelets, enabling the study of cell-released inflammatory mediators and the cross-talk between the whole inflammatory network. Importantly, we could not observe any activation of coagulation as measured by PTF1+2 upon incubation with air bubbles in plasma, underscoring the importance of experimenting in whole blood when studying this readout.

Antifoam is often used when studying air bubbles in vitro. We tested two different antifoams and found that antifoam B showed substantial adverse effects by activating complement and coagulation by increasing C3bc, C3bBbP, and PFT1+2. Thus, we designed a model system where incubation of air bubbles rather than continuously bubbling air through blood eliminated the need for antifoam. Heparin is known to interfere with complement, coagulation, and inflammation (23). Also, we have previously shown that ambient air in tubes triggers complement activation (22). Antifoam, heparin, and ambient air were avoided in our experimental model, minimizing undesired “background” activation, enabling us to study the complement, inflammation, and coagulation cascades with a higher signal-to-noise ratio than would otherwise have been possible.

Our study’s main limitation is that it was performed ex vivo, and caution should be observed in translating the data to the in vivo situation. Although whole blood is the closest available model to a physiological condition, it needs to be anticoagulated, whereby thrombin is inhibited. Furthermore, the epithelial cells with their glycocalyx are missing, and this precludes investigation of their interactions with the blood cascade systems.

If our ex vivo findings translate to in vivo conditions, we suggest that clinicians should maintain a strong focus on the detection of air emboli, such as by using echocardiography as described in a pig model (49), during high-risk procedures such as thoracic surgery, hysteroscopy, or extracorporeal blood circulation. Our findings suggest that C3 inhibition might dampen the thromboinflammatory response and could be a future approach for treatment. The recent clinical approval of the first compstatin-based C3 therapeutic, pegcetacoplan (Empaveli; Apellis Pharmaceuticals, Waltham, MA) (50, 51), opens up opportunities for applying therapeutic C3 modulation to a broad spectrum of thromboinflammatory indications in which C3 activation evokes undesirable effects, including surgery-associated venous air embolism.

In conclusion, air bubbles in lepirudin-anticoagulated human whole blood activated complement, predominantly through the alternative pathway, and subsequently triggered thromboinflammation. C3 inhibition attenuated the complement activation, hemostasis, and cytokine release, whereas C5 inhibition had only minor effects.

## Supplementary Material

Data Supplement
