# Supplementary material for: Air Bubbles Activate Complement and Trigger Hemostasis and C3-Dependent Cytokine Release Ex Vivo in Human Whole Blood
Source: J Immunol. 2021 Dec 1;207(11):2828–40. doi: 10.4049/jimmunol.2100308 (PMC8611197; doi:10.4049/jimmunol.2100308)
Supplement: Data Supplement [file JI_2100308.zip › JI_2100308_Supplemental_1.pdf]

Supplemental figure S1

Eculizumab titration in alternative pathway Wielisa and PMX53 titration in whole blood

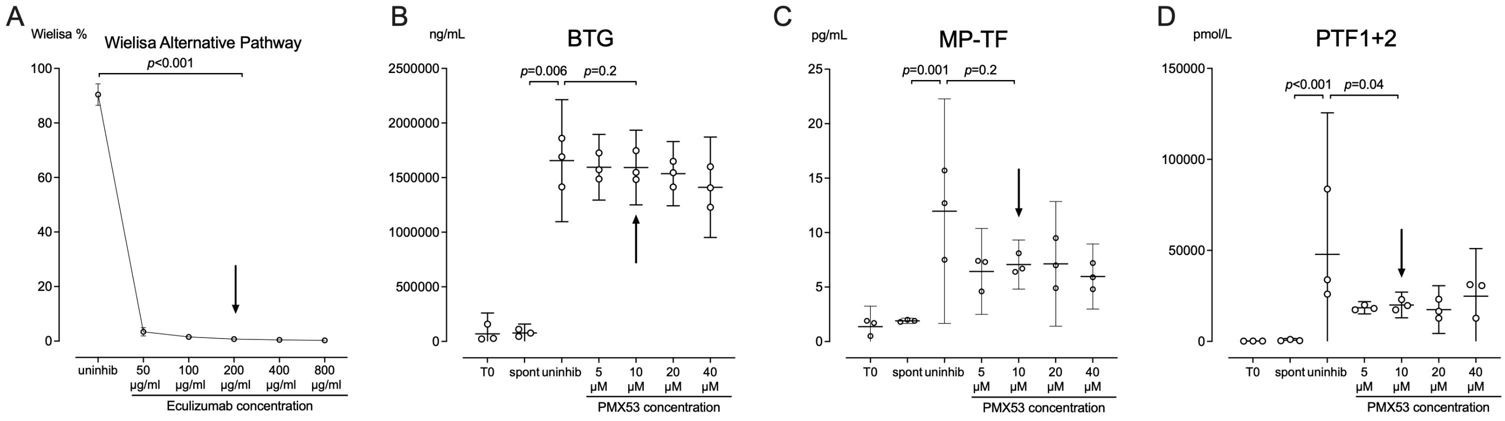

Panel A: Normal human serum was incubated without or with Eculizumab in five concentrations for five minutes at 37°C and analyzed using a Wieselab Complement system alternative pathway Wielisa (showing complement activity between 0% and 100%). Eculizumab effectively reduced the AP activity in all tested concentrations. *Panel B-D:* Lepirudin-anticoagulated human whole blood from three donors was incubated without *(spont)* or with air bubbles *(uninhib)* or with air bubbles and PMX53 in four concentrations for 180 minutes on a roller mixer at 37°C. Plasma was isolated, inactivated by EDTA, and analyzed using ELISA for BTG *(panel A)*, MP-TF *(panel B),* and PTF1-2 *(panel C)*. Incubation with air significantly increased all readouts. Incubation with PMX53 did not reduce the BTG in any tested concentration, non-significantly reduced MP-TF, and significantly reduced PTF1+2 equally in all tested concentrations. Arrow marks the concentration used in the *in vitro* whole blood air bubble study. Arrow marks the equivalent concentration used in the *in vitro* whole blood air bubble study. Graphs show means with 95% CI. *p* values were calculated using ratio paired t-test.

Supplemental figure S2

Blood gas and blood chemistry

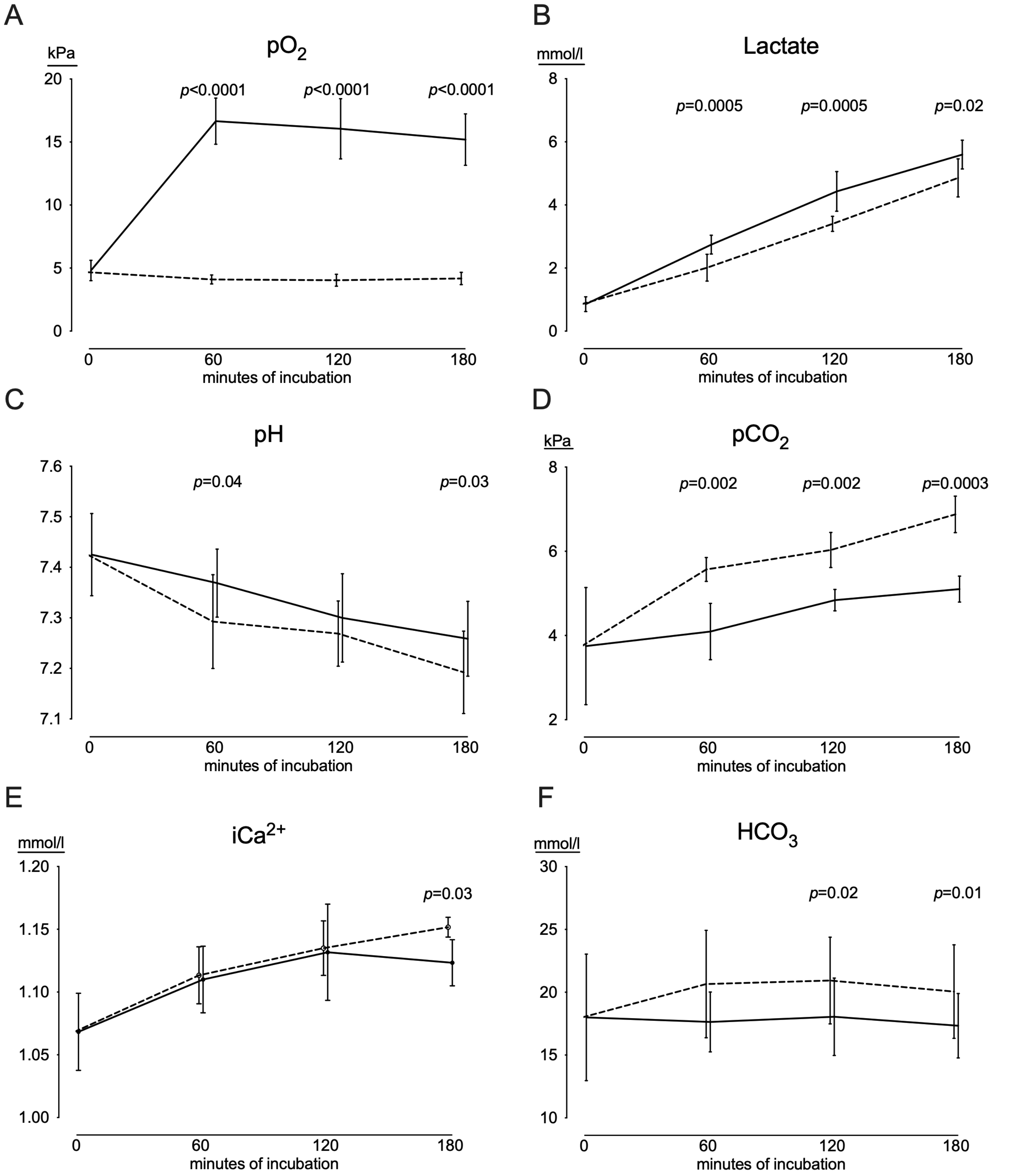

Lepirudin-anticoagulated human whole-blood from six donors was incubated for 180 minutes at 37°C on a roller mixer either in tubes without air (dotted lines) or with air bubbles added to the tubes (solid lines). pO2 (panel A), lactate (panel B), pH (panel C), pCO_2 (panel D), iCa^{2+} (panel E) and HCO_3 (panel F) sampled after 0, 60, 120, and 180 minutes of incubation. Graphs show means with 95%CI. *p*-values were calculated by multiple t-tests. Only significant *p*-values ($p<0.05$) are shown.

Supplemental figure S3

Antifoam incubations in whole blood

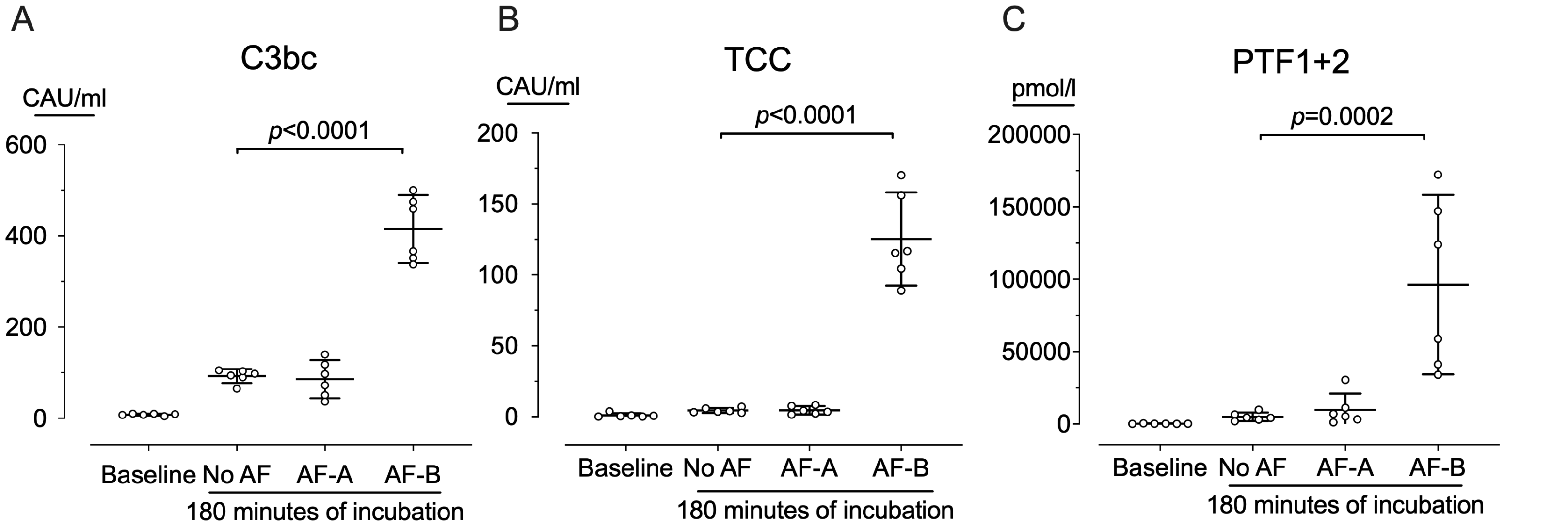

Whole blood from six donors was incubated 180 minutes on 37°C on a roller mixer in 1 mL aliquots with ambient air present in the tubes and with either no additives (*No AF*), 2 μL Antifoam A (*AF-A*), or 6 μL Antifoam B (*AF-B*). Graphs show means with 95%CI. *p*-values were calculated using RM-ANOVA with Geisser-Greenhouse correction and Fischer's exact t-test on log-transformed data. Only significant *p*-values ($p<0.05$) are shown.

Plasma air bubble incubations

Supplemental figure S4

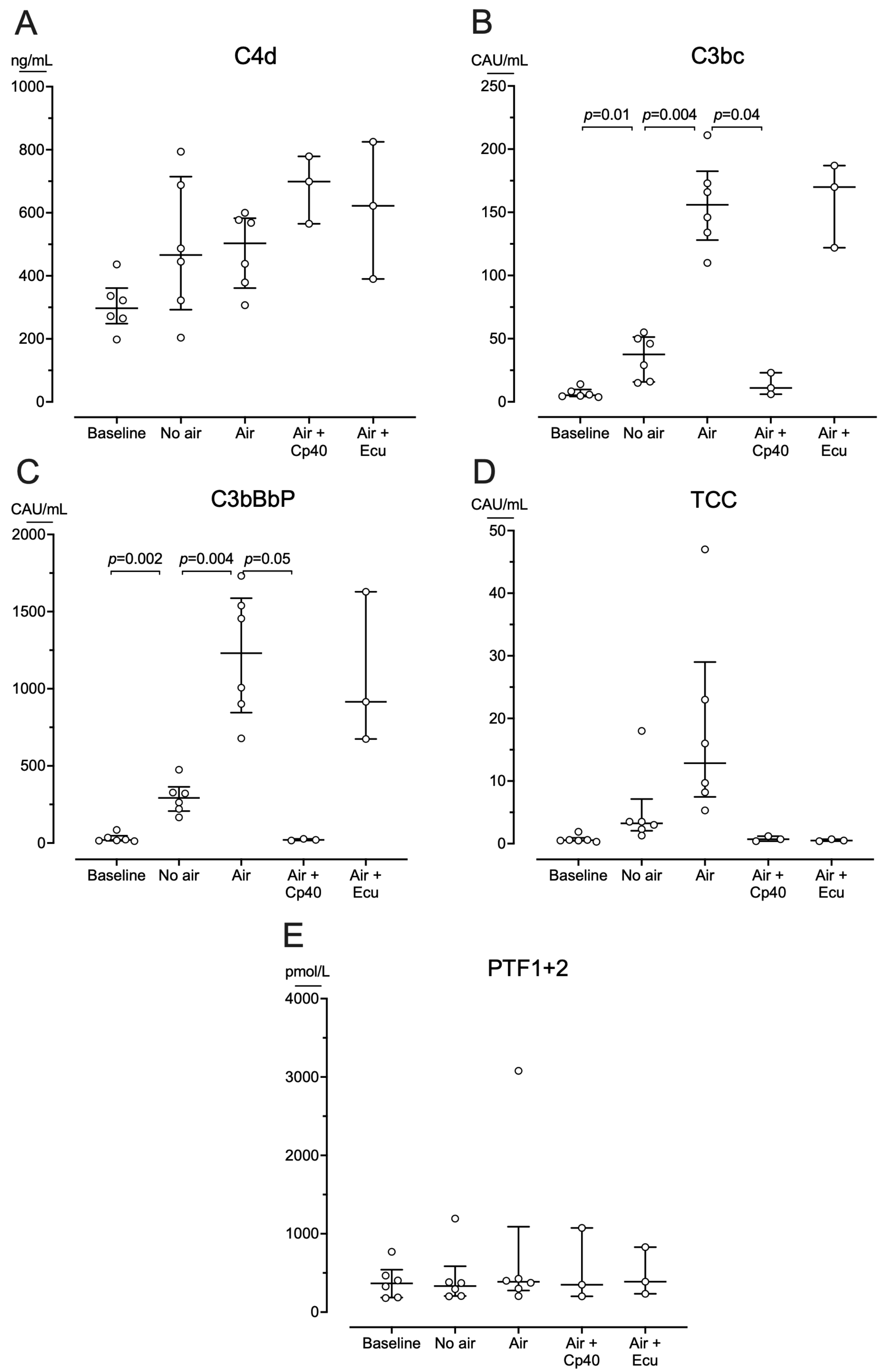

Fresh plasma from six donors was incubated for 180 minutes with either no air (n=6), air bubbles (*Air*; n=6), air bubbles and Cp40 (*Air+Cp40*; n=3) or air bubbles and eculizumab (*Air+Ecu*; n=3) and analyzed for C4d (*A*), C3bc (*B*), C3bBbP (*C*), TCC (*D*), and PTF1+2 (*E*). Graphs show means with 95% CI. *p*-values were calculated using a mixed-effects model. Only significant *p*-values ($p<0.05$) are shown.
